# Avulsion of the Flexor Digitorum Profundus Tendon During the Curettage of a Recurrent Enchondroma: A Case Report

**DOI:** 10.7759/cureus.74448

**Published:** 2024-11-25

**Authors:** Shunpei Hama, Masataka Yasuda, Makoto Fukuda, Katsuaki Onishi, Takaaki Chikugo

**Affiliations:** 1 Department of Orthopaedic Surgery, Yodogawa Christian Hospital, Osaka, JPN; 2 Department of Orthopaedic Surgery, Baba Memorial Hospital, Sakai, JPN; 3 Department of Pathology, Kindai University Faculty of Medicine, Osakasayama, JPN

**Keywords:** avulsion, calcium phosphate bone cement, distal phalanx, flexor digitorum profundus, recurrent enchondroma

## Abstract

Enchondroma rarely occurs in the distal phalanx, and avulsion of the flexor digitorum profundus (FDP) tendon in this area is also rare. We report a case of recurrent enchondroma in the distal phalanx, which required reconstruction for an accidental FDP avulsion during surgery. A 36-year-old right-handed woman visited our hospital with a suspected recurrence of enchondroma and a planned surgery. Radiographs and computed tomography revealed three translucent lesions in the distal phalanx of her left index finger. We performed curettage and calcium phosphate bone cement (CPC) grafting. During the procedure, an avulsion of the FDP tendon was found. We reattached the FDP tendon using a pull-out technique. The pathological diagnosis confirmed enchondroma. One year after the operation, she reported no pain. The range of motion (ROM) for distal interphalangeal (DIP) joint extension was 5 degrees for the right index finger and -15 degrees for the left index finger. Both ROMs of the DIP joint in flexion were 75 degrees. To the best of our knowledge, this case is notable for the use of CPC grafting and represents the third reported case of FDP avulsion due to recurrent enchondroma in the distal phalanx.

## Introduction

Enchondroma is a common benign bone tumor, accounting for 12-24% of all benign bone tumors and 3-10% of all bone tumors [1]. While enchondroma may present with pain, swelling, and pathological fracture, most cases are discovered incidentally on radiographs in asymptomatic patients. Pathological fractures often result from relatively minor injuries [2]. Hand surgeons aim to remove the tumor, and enhance bone healing so as to prevent pathological fracture [3]. Treatment options include curettage alone, curettage with autogenous bone grafting, and curettage with bone graft substitute [3]. The recurrence rate post-surgery is reported to be less than 2% [4]. The proximal phalanx is the most common location for enchondroma, followed by the metacarpals, while occurrences in the distal phalanx are rare, as is avulsion of the flexor digitorum profundus (FDP) in this area [5]. To date, 15 cases of FDP avulsion due to enchondroma have been documented [2,4-16]. Surgical repair options for FDP avulsion include pull-out techniques [4,5,7-9,11,12,14,15], open reduction and internal fixation (ORIF) [6,10], intraosseous wiring (IOW) [13], and intraosseous fixation (IOF) [16]. We present a case of recurrent enchondroma in the distal phalanx that required reconstruction due to an accidental FDP avulsion during surgery.

## Case presentation

**Figure 1:**
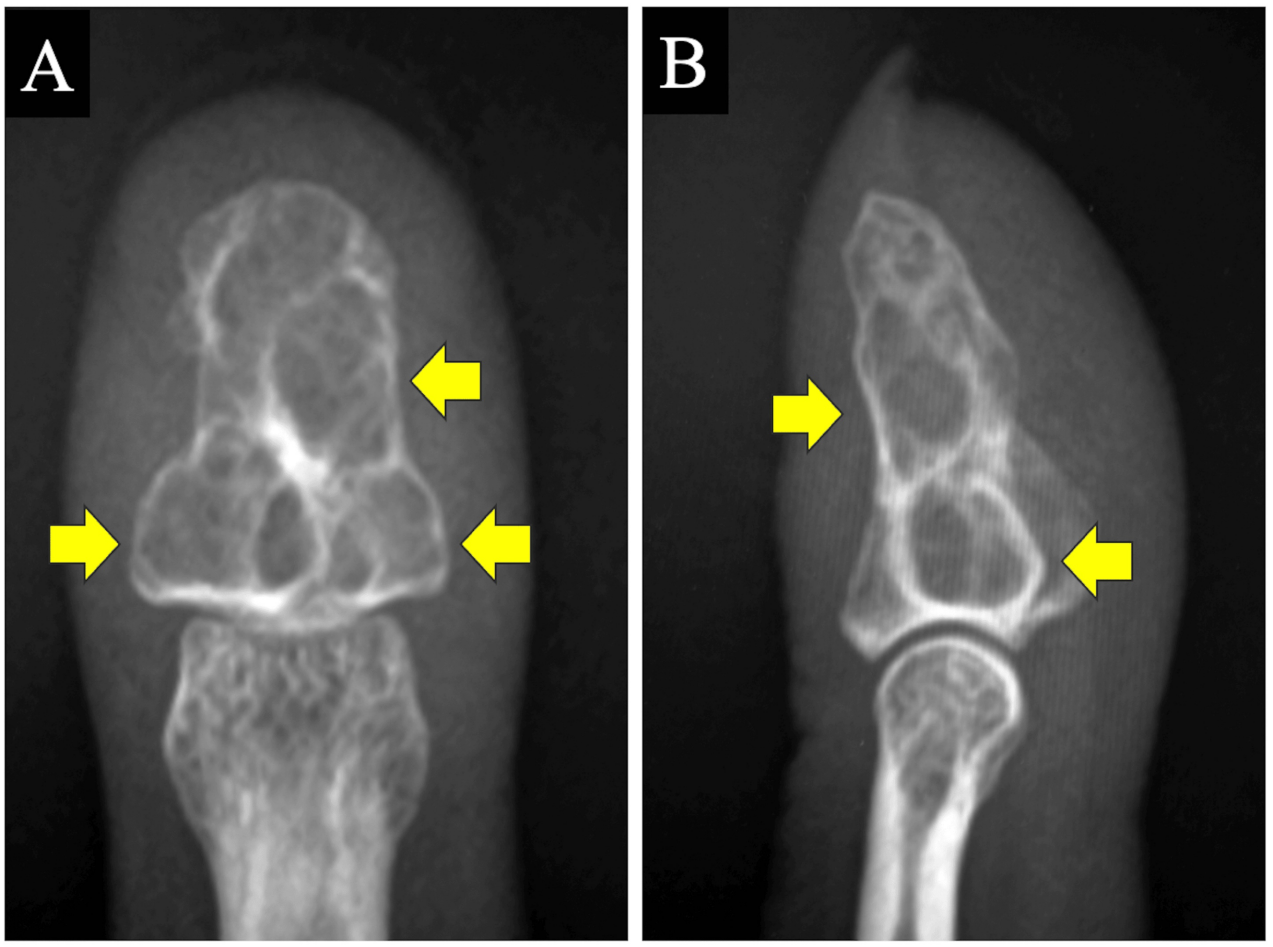
Preoperative radiographs of the left index finger. (A) Posteroanterior view; (B) lateral view (arrows indicate translucent lesions).

**Figure 2:**
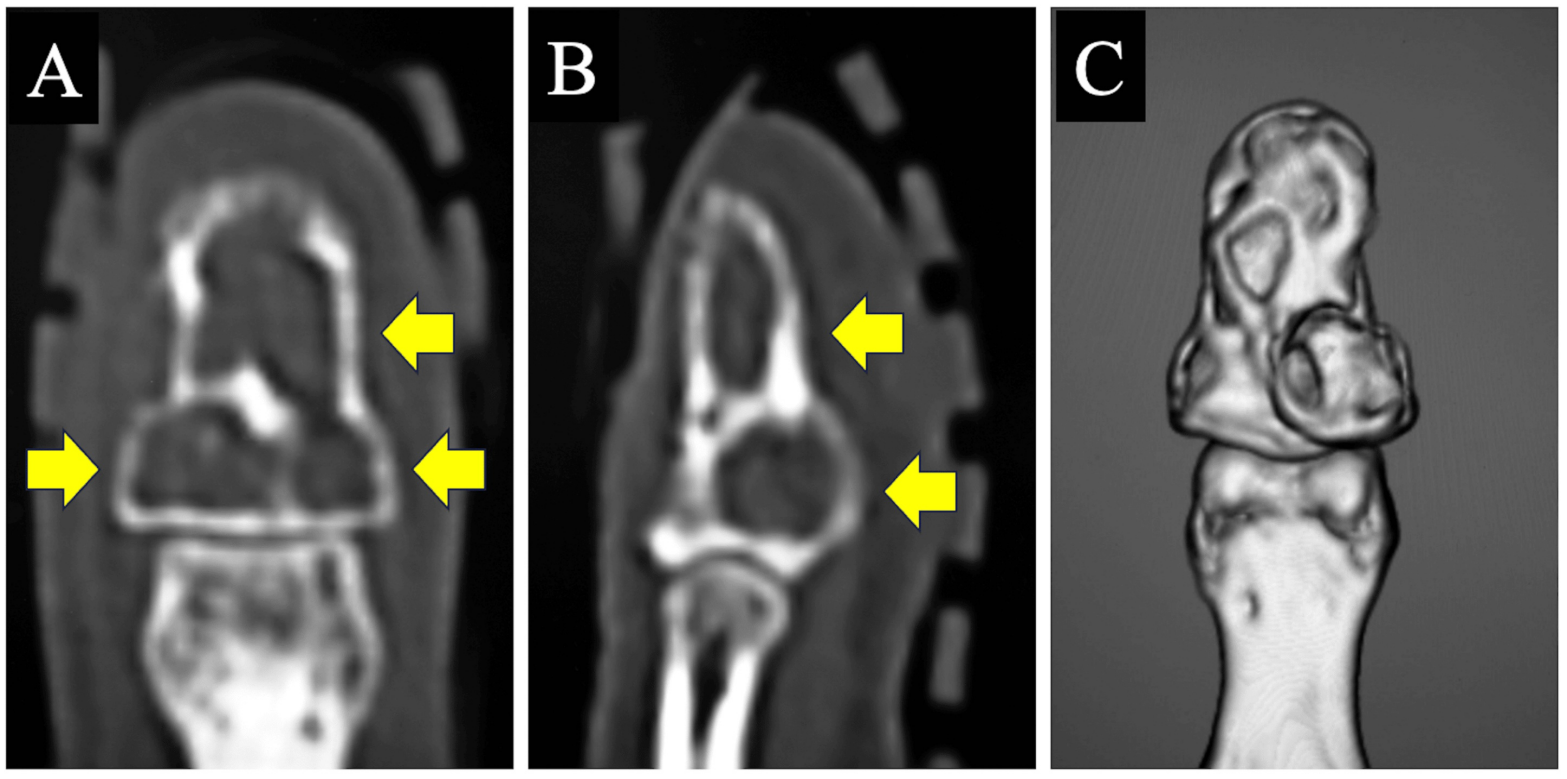
Preoperative computed tomography of the left index finger. (A) Coronal image; (B) sagittal image (arrows indicate translucent lesions); (C) three-dimensional image.

A 36-year-old right-handed woman presented with pain in the distal phalanx of her left index finger. She had undergone surgery for enchondroma in the distal phalanx 14 years earlier, involving curettage and placement of a bone graft substitute. However, her pain worsened about three months earlier, and upon visiting another hospital, a recurrence of the tumor was suspected, prompting her referral to our facility. Physical examination revealed slight swelling of the volar terminal phalanx and mild knocking pain. The previous incision was radial. Radiographs and computed tomography (CT) identified three translucent lesions in the distal phalanx of her index finger (Figures 1, 2). Suspecting recurrence, we planned surgical intervention.

**Figure 3:**
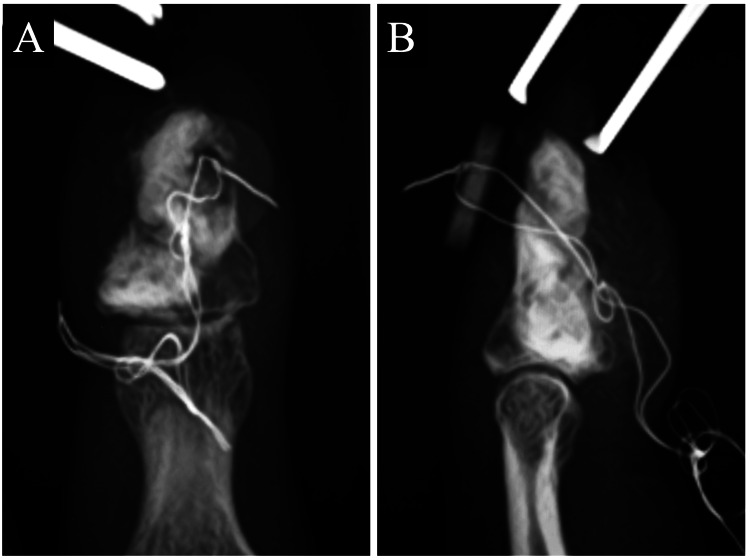
Postoperative radiographs of the left index finger. (A) Posteroanterior view; (B) lateral view.

**Figure 4:**
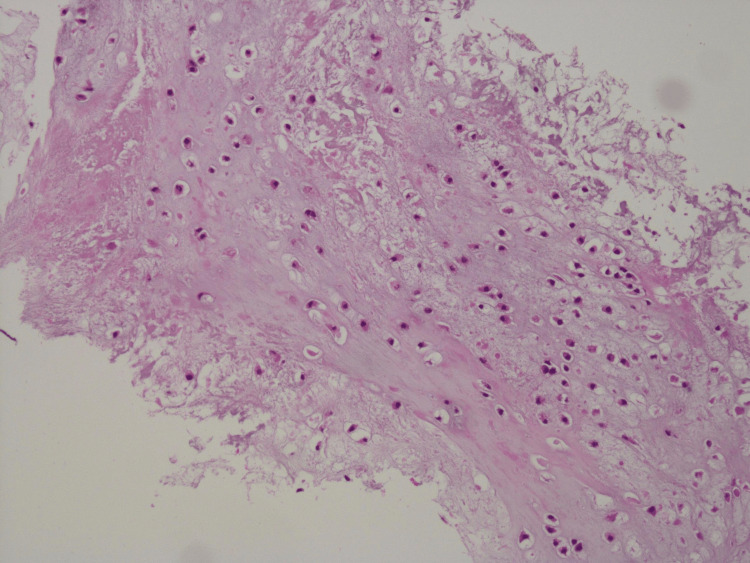
Histopathological examination of the tumor stained with H&E. The nuclear morphology and stromal matrix were hyaline cartilage-like, and the nuclei were basically oval, poorly hyperplastic, and within the benign range, although nuclear density was somewhat high in some areas. There were no aggressive findings suggestive of malignant transformation.

**Figure 5:**
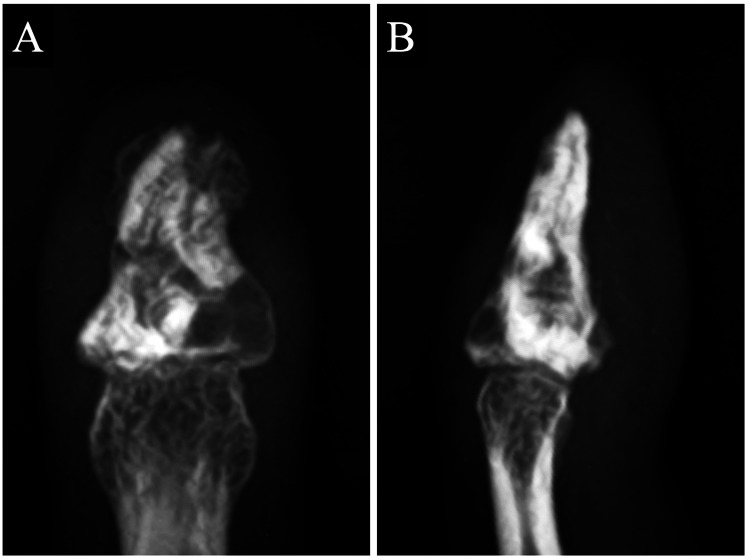
Radiographs of the left index finger one year after the surgery. (A) Posteroanterior view; (B) lateral view.

Surgery was performed under Oberst’s block anesthesia with a central longitudinal incision on the palmar aspect of the distal phalanx. The tumor’s appearance was consistent with enchondroma, occupying nearly the entire distal phalanx. The palmar cortex was thinned and chipped away during curettage. Calcium phosphate bone cement (CPC) paste Biopex-R (Hoya Technosurgical Co., Tokyo, Japan) was injected to fill the tumor cavity [3]. However, an FDP tendon avulsion was noted. The FDP tendon avulsion was thought to be due to curettage in this surgery because she could preoperatively flex the distal interphalangeal joint (DIP). An attempt to reattach the tendon with an anchor (Micro-Mitek, DePuy Mitek, Raynham, USA) failed due to insertion difficulties. The tendon was instead reattached using a pull-out technique (Figure 3). Post-surgery, a dorsal plaster splint was used to immobilize the hand and wrist in a slightly flexed position. The tumor was pathologically confirmed as enchondroma (Figure 4). Three weeks post-surgery, the splint was removed, and six weeks after the surgery, the pull-out wire was removed, allowing an active range of motion (ROM). One year postoperatively, she reported no pain, and her DIP extension ROM was 5 degrees for the right index finger and -15 degrees for the left. Flexion ROMs were both 75 degrees. Follow-up radiographs showed no recurrence, with partial absorption of the bone graft substitute (Figure 5).

## Discussion

We conducted curettage and CPC grafting, followed by FDP reattachment using a pull-out technique for this recurrent enchondroma, which required FDP reconstruction after an accidental avulsion during surgery. To date, 15 cases of FDP avulsion secondary to enchondroma have been reported [2,4-16]. Avulsions most commonly occur in the ring finger (six cases) [2,4,6,12,14,16], followed by the little finger (five cases) [7,9,11,12,15] and the middle finger (four cases) [5,8,10,13]. The frequent avulsion in the ring finger may be attributed to a common flexor muscle belly of the profundus to the middle, ring, and little fingers, by the intertendinous connections between the extensor tendons, and by a weakness of its insertion [8]. To the best of our knowledge, this is the first case of FDP avulsion in the index finger due to enchondroma. This might be because the present case was a recurrent case and the bone cortex at the insertion of the FDP tendon was too thin to withstand the curettage. To avoid complications, it is important to perform curettage carefully so as not to cause FDP avulsion. Even if intraoperative FDP avulsion does not occur, it may be an option to reinforce FDP using a pull-out technique to avoid postoperative avulsion. Only two other cases of FDP avulsion due to recurrent enchondroma have been reported [4,5], making this the third.

The average recurrence period of the previously reported cases was 17.5 years, similar to the present case. A possible cause of tumor recurrence is leftover tumor cells. The two recurrent cases previously reported were treated with the pull-out technique for the FDP avulsions, which had happened before surgical treatment of the enchondroma recurrency and autogenous cancellous bone grafting [4,5]. The present case was unique in that the FDP avulsion occurred intraoperatively and CPC grafting was performed. The average age of the previously reported patients was 39.7 years, similar to our patient’s age. There was a female predominance (six males and nine females), and our patient was also female. Ten of the cases employed the pull-out technique for FDP reattachment [4,5,7-9,11,12,14,15], while two used ORIF [6,10], and two others underwent IOW and IOF [13,16]. The remaining patient had to bolster the suture [2]. Twelve cases involved autogenous cancellous bone grafting [4-10,12-15], though others did not [2,11,16]. In our case, we used CPC grafting, avoiding the disadvantages of autogenous grafting, such as donor site morbidity, increased bleeding, extended surgery time, temporary mechanical weakness [3], and contamination of the donor site by tumor cells. Although our patient could flex the DIP joint preoperatively, the FDP avulsion occurred unexpectedly during curettage. Anchor fixation was attempted but unsuccessful, leading to pull-out reattachment after CPC grafting. Surgeons should be prepared for accidental FDP avulsion when performing curettage for enchondroma in the distal phalanges of the finger.

## Conclusions

This case is notable for the use of CPC grafting, and to the best of our knowledge, this is the third reported case of FDP avulsion secondary to recurrent enchondroma in the distal phalanx. Hand surgeons should be prepared for the possibility of accidental FDP avulsion during curettage for enchondroma in the distal phalanges of the finger. To avoid the avulsion, it is important to perform curettage carefully. Even if intraoperative FDP avulsion does not occur, it may be an option to reinforce FDP with a pull-out technique to avoid postoperative avulsion.
